# A New Test for Diabetis

**Published:** 1861-03

**Authors:** E. C. Bidwell


### *A New Test for Diabetis.* By E. C. Bidwell, M. D.

The only tests for glucoscuria which I have hitherto found satisfactory—fermentation—involves a delay which is often exceedingly annoying, and sometimes fatal to a satisfactory and seasonable diagnosis. Those founded upon the reduction of metalic oxides, besides being complicated and inconvenient for clinical use, are liable to various fallacies. A better test than any I have seen described, seemed to me a desideratum—one which should be delicate and conclusive, and at the same time ready and convenient. Moved by this sense of a want, to experiment for a new process, I have discovered one which seems to meet fully the needs of the case; one, which, if it be not pre-eminently scientific, is nevertheless facile and reliable. For the benefit of any others who may have felt the same want, I herewith communicate the result of my investigations.

Technically described, it is simply the conversion of the saccharine element of diabetic urine into *caramel* by heat. My mode is this: Upon a clean slip of tinned iron, place one or two drops of the suspected material, and hold it over a spirit lamp: the fluid will speedily evaporate, leaving, if the process be arrested at that point, scarcely a trace upon the metallic surface. Continue the application of heat; in a few moments after the desication is complete, a spot of an inch or so in diameter, over which the drop had spread with the first ebulition, will gradually assume a rich reddish-brown color, with a brilliant lustre, as if coated with a film of varnish or Japan lacquer. A strong heat produces a darker color, but the lustre continues till the heat becomes sufficiently intense to decompose the substances.

This experiment has succeeded perfectly in my hands, when the urine on trial, previously known to contain glucose, was of specific gravity less than 1030, and still further reduced by the addition of three or four times as much of water. It is thus proved to be a delicate test. I suppose it to be conclusive, also, for I have never yet found any other constituent of urine, normal or abnormal, capable of producing anything at all like the same appearance under the same treatment. The nearest approach is this: some samples of urine not diabetic, when treated in this way, leave a faint, dull, yellowish stain, easily distinguished from caramel by its paler color, and the entire absence of lustre. I need scarce add, that a solution of sugar, not diabetic, exhibits almost exactly the same reaction.

With the augmented interest attached to glucosuria, since, besides being a leading feature of a most intractable, but fortunately rare, disease, it is found symptomatically associated with several other diseases and injuries, an increased facility for its detection is almost a necessity of the profession. I trust they will find it in the simple and beautiful experiment above described.—*Southern Medical & Surgical Journal.*

